# Curcumin Attenuates Glyphosate-Induced Mammary Toxicity via Suppression of ER Stress and the TNFα/MAPK/STAT3 Axis

**DOI:** 10.3390/antiox15070893

**Published:** 2026-07-19

**Authors:** Yonglong He, Hanbing Yan, Zesheng Gan, Ziwei Cheng, Binyun Cao, Jiangang Wang, Xiaopeng An

**Affiliations:** College of Animal Science and Technology, Northwest A&F University, Yangling, Xianyang 712100, China; heyonglong96@nwafu.edu.cn (Y.H.);

**Keywords:** glyphosate, curcumin, transcriptome, toxicity, oxidative stress, apoptosis

## Abstract

Curcumin, a natural polyphenol with well-established antioxidant and anti-inflammatory properties, was evaluated for its protective effects against glyphosate (GLY)-induced mammary toxicity. Pregnant mice received GLY (50 or 250 mg/kg/day) with or without curcumin (150 mg/kg/day) by oral gavage; GLY dose-dependently disrupted alveolar architecture, caused marked inflammatory infiltration, and downregulated tight-junction proteins. In parallel, goat mammary epithelial cells (GMECs) were treated with GLY (1 or 7 mM) and curcumin (1 µM) to dissect acute cellular stress pathways. These complementary models address distinct aspects of GLY toxicity: the in vivo system reflects defined oral exposure, whereas the in vitro setting employs high concentrations to probe mechanistic events. Transcriptomic profiling combined with functional assays demonstrated that GLY triggered oxidative stress, endoplasmic reticulum stress, and intracellular Ca^2+^ overload, activated the TNFα/MAPK axis, suppressed STAT3 phosphorylation, and ultimately promoted apoptosis. Curcumin co-treatment alleviated these alterations at both structural and molecular levels. These findings indicate that curcumin can combat GLY-induced mammary injury by restoring cellular homeostasis and inhibiting apoptotic and inflammatory signaling, thereby protecting breast health.

## 1. Introduction

Due to the rapid growth of the agricultural economy, pesticides have become widely employed in various fields such as agriculture, horticulture, and forest planting [1]. Glyphosate (GLY), also referred to as N-(phosphonomethyl) glycine, serves as the active component of a highly prevalent herbicide used for weed control in agriculture [2,3]. It is frequently found in soils, plants, water, air, humans, and animals due to its extensive use [4,5].

The mode of action of glyphosate is linked with its potential to inhibit the shikimic acid pathway, which is present in bacteria, plants, fungi, and protozoa, but not in mammals [6,7]. Multiple reports and regulatory authorities, such as the European Food Safety Authority, concluded that GLY was fairly safe for human consumption [8,9]. However, there has been some debate surrounding this viewpoint as numerous studies have revealed the possible health hazards associated with GLY. Studies have shown that GLY has been found to have adverse effects on the kidney and liver even at concentrations less than the acceptable daily use [10]. There is a strong correlation between the doses of GLY exposure and the stages of liver fibrosis in patients [11]. A liver panel test reveals aberrant liver function in rats and mice exposed to GLY, while a biochemical study identifies dysregulated lipid metabolism [12,13]. Human cell lines HepG2 and MDA-MB453-kb2 were exposed to sub-agricultural concentrations of GLY-based herbicides, which caused DNA damage and endocrine imbalances in vitro [14]. Glyphosate at 10^−12^ to 10^−6^ M promotes the growth of human hormone-dependent breast cancer T47D cells by binding to estrogen receptors [15]. Moreover, adjuvants improved the solubility of GLY in water and its assimilation by plants is frequently detected in the environment and has been found to cause cellular toxicity [16,17]. The degradation mechanism of GLY in mammals remains unclear; however, animal and human subjects exposed to GLY were able to detect its metabolites, like aminomethylphosphonic acid (AMPA) [18,19]. The primary glyphosate metabolite, AMPA, has also been implicated in the emergence of toxicity [20]. In response to these concerns, the International Agency for Research on Cancer reclassified GLY as a probably carcinogenic to humans [21]. Its application has also been restricted or completely banned in almost 20 countries including Thailand, Belgium, France, and Italy [22].

Recently, oxidative stress (OS) has become one of the most significant issues in the field of pesticide toxicology [23]. DNA, lipids, and proteins within cells can be damaged and apoptotic pathways can be triggered by OS caused by an excess of ROS production or inadequate antioxidant defense (especially in the elderly) [24]. Cell death, a consequence of continuing cell damage, has a significant role in the emergence of diseases in various tissues and organs. Apoptosis and necrosis are the two primary forms of cell death, and they are distinguished by their morphological characteristics, mechanisms, and physiological and pathological functions [25]. Furthermore, cell death during autophagy demonstrates multiple biochemical characteristics similar to apoptosis. Environmental stressors, such as pesticides, have a role in the development of neurological conditions by triggering inflammation, oxidative stress, and ultimately leading to cell death.

Thus, it is important to consider the potential risks to animal and human health from the accumulation of glyphosate residues. However, the concentrations of these residues may be low, and their chronic exposure through water and food consumption could pose a concern, especially considering the widespread use of glyphosate on a large scale [26,27]. The presence of glyphosate in the urine and organs of a large number of farm animals and farmers has been confirmed [28,29]. Furthermore, urine samples from 60 to 80% of the general public in the United States contained residues, with maximal concentrations of 233 μg/L and medium concentrations ranging from 2 to 3 μg/L. Residues were found in the urine of 44% of the European population, with average (1 μg/L) and maximum amounts (5 μg/L) [30].

Recent results on glyphosate environmental contamination indicate that the toxicity of severe exposure to lower amounts of this substance may be more significant than acute toxicity. Therefore, there has been a significant rise in the number of articles demonstrating the long-term harmful effects of glyphosate on animals and humans. There is growing concern about the possible adverse effects on animal and human health that could result from prolonged exposure to glyphosate. Recently, glyphosate has attracted more interest nationally or internationally from regulatory agencies, as well as the scientific community. The reclassification of glyphosate by the International Agency for Research on Cancer (IARC) of the World Health Organization (WHO) as probably carcinogenic to humans in 2015 was based on research regarding its chronic adverse effects. However, the European Union evaluation and the latest joint assessment by the Food and Agriculture Organization of the United Nations (FAO)/WHO have not validated IARC’s conclusion [31]. This underscores the presence of substantial divergence among regulatory bodies, along with the imperative to achieve consensus and revise safety guidelines for glyphosate to overcome public and environmental health.

Curcumin, an anti-cancer, anti-inflammatory, and yellow phenolic active ingredient found in turmeric, is extracted from the rhizome of *Curcuma longa* L. [32,33,34]. Curcumin, a natural pigment and functional food ingredient, finds extensive application in the food and beverage industry [35]. Curcumin is solid in the acidic environment of the stomach but insoluble in water due to its lipophilic nature. Curcumin has a multifactorial effect through its interaction with a diverse array of cell signaling molecules, including but not limited to proinflammatory cytokines, apoptotic proteins, transcription factors, enzymes, and proteins. Curcumin is GRAS-approved for food use under specific conditions: concentration limits (0.5–100 mg per 100 g of food) vary by GRAS number for ingredients, supplements, or flavorings, excluding infant formula and USDA foods. EU limits intake to ≤3 mg/kg body weight. Curcumin should be stored away from light, heat, and iron to maintain its stability Besides inhibiting preadipocyte differentiation in vitro, curcumin inhibits the expression of inflammatory cytokines stimulated by TNFα in adipocytes [36]. Curcumin synergistically sensitizes multidrug-resistant lung cancer cells to doxorubicin through ferroptosis-associated oxidative stress, with curcumin administered at 40–70 μM [37]. The efficacy of curcumin in acute and chronic models of inflammation has been supported by accumulating evidence. Acute lipopolysaccharide (LPS) induced inflammatory damage is reduced in mice post-curcumin administration after experimental traumatic brain injury [38]. Curcumin has the potential to disrupt the level and recruitment of KC, IL-1β, MIP-2, and MIP-1α in macrophages and colonic epithelial cells (CECs) stimulated by LPS [39]. Therefore, it has implications for the development of inflammatory bowel disease (IBD). Despite the accumulating evidence on the anti-inflammatory properties of curcumin, there are still significant gaps in the understanding of its mode of action.

Recently, the introduction of genetically engineered glyphosate-resistant crops has facilitated the global dissemination of glyphosate applications [40]. The formulations and derivatives of glyphosate have garnered extensive research attention due to their potential implications for animal health and environmental safety. A comprehensive understanding of the mechanisms underlying glyphosate exposure in the mammary glands of mammals is therefore urgently needed to address the current gap in knowledge regarding its effects on this tissue. In this study, we established a glyphosate (GLY) exposure model in pregnant mice and an in vitro model using goat mammary epithelial cells (GMECs). Importantly, the doses used in vivo (50–250 mg/kg/day) and the concentrations applied in vitro (1–7 mM) differ substantially, reflecting their distinct experimental purposes: the in vivo model was designed to assess systemic mammary toxicity at high exposure levels, whereas the in vitro model employed acute high concentrations to dissect cellular stress pathways rather than to directly mimic environmental exposure conditions. Through histopathological examination combined with transcriptome sequencing, we characterized the damage to the mammary glands induced by high-dose glyphosate (250 mg/kg/day in vivo; 7 mM in vitro) and identified a protective mechanism mediated by curcumin (150 mg/kg/day in vivo; 1 μM in vitro). These findings not only provide new mechanistic insights into glyphosate-induced mammary toxicity but also suggest an accessible strategy for mitigating the adverse effects of glyphosate using curcumin.

## 2. Materials and Methods

### 2.1. Animals and Study Design

All animal treatments involving mice were reviewed and approved by the Animal Ethics Committee of Northwest A&F University (Xianyang, China). A total of 40 female and 20 male healthy SPF Kunming mice (7 weeks of age) were purchased from the Animal Experiment Center at Northwest A&F University. Mice were kept on a 12 h light/dark cycle at 23 °C with ad libitum access to food and water and group-housed, with 4 per cage. All mice were allowed to acclimate for a week before treatment.

Female mice were mated with males at a 2:1 ratio. Pregnant females of comparable body weight were randomly assigned to four groups (*n* = 8 per group). Beginning on gestational day 15, mice received daily oral gavage for the entire treatment period, which continued through parturition until the day before sample collection. The four groups were: control (control, 0 mg/kg/day), low-dose glyphosate (L-GLY, 50 mg/kg/day), high-dose glyphosate (H-GLY, 250 mg/kg/day), and H-GLY combined with curcumin (H-GLY + Cur, 250 mg/kg GLY + 150 mg/kg Cur). Glyphosate (CAS 1071-83-6, Macklin, Shanghai, China), used in its water-soluble form, was dissolved directly in ultrapure water. Due to the low aqueous solubility of curcumin (CAS 458-37-7, Macklin, Shanghai, China), it was initially dissolved in dimethyl sulfoxide (DMSO) and then diluted in normal saline for in vivo gavage or in culture medium for in vitro experiments, with the final DMSO concentration maintained below 0.1% (*v*/*v*) in all instances. The gavage volume was 0.2 mL per 10 g body weight. All dams delivered normally. Animals that met pre-established exclusion criteria were removed, leaving a final sample size of *n* = 6 dams per group for analysis. The doses of GLY and Cur were selected based on previous studies [41,42]. On lactation day 10, approximately 24 h after the last gavage, the mice were anesthetized with pentobarbital, and mammary tissue samples were collected for further analysis.

### 2.2. Cell Culture and Treatment

Tissues from the mammary glands of 3-year-old Guanzhong dairy goats were obtained during the lactation period at peak. The samples were then stored at 4 °C in PBS solution containing (100 μg/mL) streptomycin/penicillin. GMECs were isolated from lactating goat mammary gland tissue as described by Wang, using collagenase digestion and differential centrifugation [43]. These cells were allowed to grow in DMEM/F12 media (HyClone, Logan, UT, USA) supplemented with fetal bovine serum (FBS, 10%) and incubated in a 5% CO_2_ at 37 °C. The media was changed, and enzymatic detachment of the cells was carried out via trypsin. This was followed by the transferring of the cells to a new culture when they reached 80% confluency.

### 2.3. RNA-Seq Analysis

Mammary samples were stored in RNase-free cryovial, quick-frozen in liquid nitrogen. Total RNA was extracted from mammary samples and GMECs using the TRIzol method. Next, the samples were sent to Beijing Novogene Biotechnology (Beijing, China) for high-throughput transcriptome sequencing. RNA quality was assessed with an Agilent 5400 Bioanalyzer (Agilent Technologies, Santa Clara, CA, USA) and a Nanodrop spectrophotometer (Thermo Fisher Scientific, Waltham, MA, USA). Libraries were prepared with the NEBNext Ultra RNA Library Prep Kit (New England Biolabs, Ipswich, MA, USA) for Illumina and sequenced on an Illumina NovaSeq platform (150 bp paired-end reads). Raw reads were quality-filtered using Fastp (v0.19.7; parameters: −g −q 5 −u 50 −n 15 −l 150). Clean reads were aligned to the mouse reference genome (Ensembl GRCm38.p6, GCA_000001635.8) and the goat reference genome (Ensembl ARS1.2, GCA_001704415.1) with STAR, and gene-level read counts were generated by featureCounts (Subread package v2.0.1). Differential expression analysis was performed using DESeq2 (R package v1.34.), with thresholds of adjusted *p* < 0.05 and |log2(fold change)| ≥ 1. GO and KEGG enrichment analyses were conducted with the clusterProfiler package (R package v4.2.2).

### 2.4. Histopathology

The paraffin-embedded tissue sections need to be first de-waxed with xylene, and then hydrated with a series of alcohol gradients to allow water-soluble dyes to enter the tissue. Histological changes were evaluated in a blinded manner using a semiquantitative scoring system (0–3), with 0 = normal histology, 1 = mild, 2 = moderate, and 3 = severe alterations. Scoring was based on the degree of alveolar structural disruption, epithelial vacuolation, and interstitial inflammatory cell infiltration [44].

### 2.5. Cytotoxicity Assay

The cytotoxicity of glyphosate on GMECs was evaluated using the Cell Counting Kit-8 (CCK-8; Sigma, Shanghai, China). Cells were placed on 96-well plates with 1 × 10^4^ cells/0.1 mL/well. They were treated with various (1, 3, 5, 7, 8 or 10 mM) concentrations of glyphosate and (0.3, 0.6, 1.0, 1.2, 1.5 or 2.0 uM) curcumin for 24 h. After that, 10 μL of CCK8 was added into each well, followed by 4 h of incubation at 37 °C in the dark. The absorbance of the suspension was quantified at 450 nm via a microplate reader (BioTek, Winooski, VT, USA) [43]. Each experiment was independently replicated three times.

### 2.6. Hoechst 33258 Staining Assay

Apoptosis was assessed using the Hoechst 33258 kit (Solarbio, Beijing, China), following the manufacturer’s instructions. After exposing GMECs to L-GLY (1 mM GLY) and H-GLY (7 mM GLY) on 6-well plates, they were placed in a 4% paraformaldehyde (PFA) solution for 30 min and then rinsed (thrice) with PBS. Hoechst 33258 dye was used to stain these cells for 10 min. All the respective changes including nuclear morphological alterations were observed under a fluorescence microscope.

### 2.7. ROS Detection

Intracellular ROS oxidize non-fluorescent DCFH to fluorescent DCF, which emits green fluorescence. Thus, a DCFH diacetate kit (Beyotime, Shanghai, China) was employed to measure the levels of ROS in cells. Cells were loaded with DCFH-DA (DCFH-DA was dissolved in DMSO to a 10 mM stock solution and diluted 1:1000 in serum-free culture medium to a final working concentration of 10 μM) for 20 min at 37 °C in the dark, washed three times with PBS, and immediately visualized under a fluorescence microscope (XDS-1). Micrographs were acquired at consistent exposure settings, and the integrated fluorescence intensity per cell was quantified using ImageJ 1.54 (NIH, Bethesda, MD, USA) software with background subtraction.

### 2.8. ER Staining and Ca^2+^ Level Detection

The intracellular concentration of calcium was quantified via Ca^2+^ assay kits (Beyotime, Shanghai, China) as per the given instructions. The cells were exposed to different doses of glyphosate and curcumin, incubated with a Fluo-4 AM probe, rinsed three times with PBS, and immediately visualized under a fluorescence microscope (DMi8, Leica Microsystems, Wetzlar, Germany). ER-Tracker Red (Beyotime, Shanghai, China) was used to capture fluorescence images of the ER. Briefly, treated cells were kept in the dark at 37 °C for 20 min in 2 mM ER-Tracker staining buffer. After washing with PBS, the cells were immediately visualized under a fluorescence microscope . The intensity of fluorescence was determined using ImageJ 1.54 (NIH, Bethesda, MD, USA) software. Fluorescence intensity was quantified using ImageJ 1.54 software by outlining individual cells or regions of interest, and the integrated density after background subtraction was recorded to represent the fluorescence distribution.

### 2.9. Annexin V-FITC/PI Staining

GMECs were cultured in 6-well plates. Once the cells were confluent about 80%, they were preconditioned with glyphosate and curcumin at different doses. Next, treated cells were trypsinized and rinsed with PBS. They were then mixed with binding buffer to form a suspension. In this suspension, 5 μL of Annexin V-FITC and 10 μL of propidium iodide (PI) were added and allowed to incubate at 25 °C for 5 min in the dark. Cell apoptosis was detected by flow cytometry (BD FACSAria™ III, BD Biosciences, San Jose, CA, USA), and data was analyzed via FlowJo V11 software (FlowJo, LLC, Ashland, OR, USA).

### 2.10. Analysis of RT-PCR

Total RNA was collected from mammary and GMECs using a TRIzol kit (TaKaRa, Dalian, China) according to the manufacturer’s protocols. RNA was reverse-transcribed to cDNA with a Reverse Transcription Kit (TaKaRa, Dalian, China) following the manufacturer’s protocol. The PCR primers used for RT-qPCR are shown in Table 1. β-actin was used here as the reference gene.

### 2.11. Western Blot

The total content of protein was isolated from cells via RIPA lysis reagent (Biotek, Beijing, China) as per the given instructions. For the quantification of protein concentration, a BCA assay reagent was employed (Beyotime, Shanghai, China). A PVDF membrane (blot) (Merck Millipore, Burlington, MA, USA) was used to transfer the proteins after their separation via 12% SDS–PAGE. The blot was blocked for 2.5 h at 25 °C in a buffer consisting of nonfat dried milk (5%) and TBST with 0.1% Tween 20. The blot was then incubated at 4 °C for 24 h with the respective primary antibodies. They were then kept at 4 °C for 2 h with secondary antibodies. The bands were observed via a chemiluminescent reagent (Amersham ECL, Cytiva, Marlborough, MA, USA) and captured via a gel imager (GenoSens 2100, Clinx, Shanghai, China). These bands were semi-quantified via ImageJ 1.54 software. The antibodies used in this experiment are shown in Table 2.

### 2.12. Statistical Analysis

Data were statistically examined via GraphPad Prism 9 (GraphPad Software, San Diego, CA, USA). All experiments were repeated at least three times, and the differences among means were evaluated using one-way ANOVA (ANOVA). The data are expressed as the mean ± standard error of the mean (SEM), and statistically significant differences were identified at a *p* value < 0.05.

## 3. Results

### 3.1. Histopathology of the Mammary Gland

**Figure 1:**
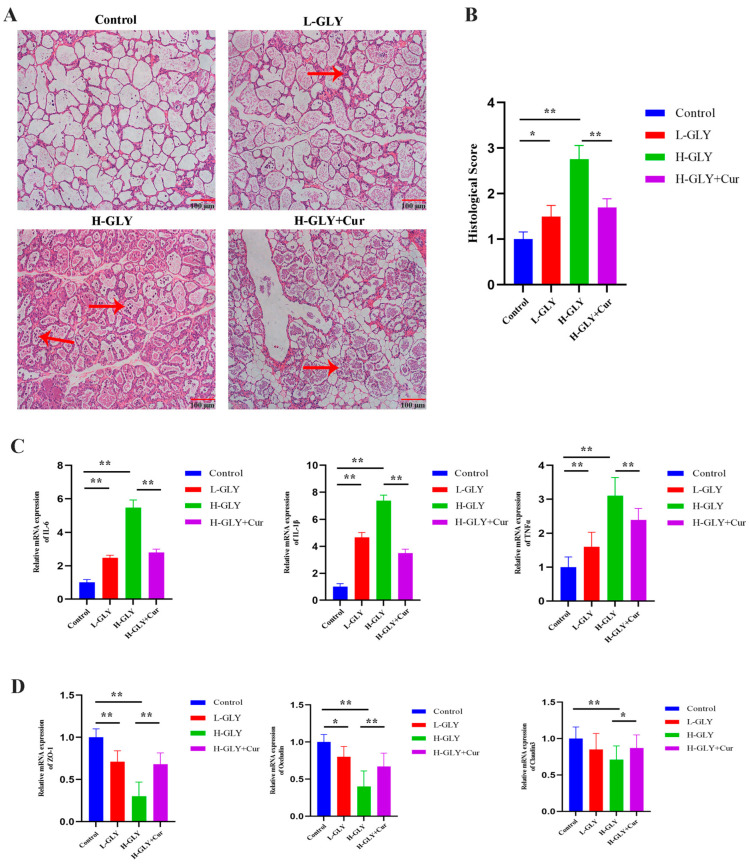
Histomorphology and barrier of mammary gland in pregnant mice. (**A**) HE staining of the mammary, the red arrowheads indicate inflammatory cell infiltration. Scale bar is 100 μm. (**B**) Histological score. (**C**) Relative mRNA expression of IL-6, IL-1β and TNFα. (**D**) Relative mRNA expression of ZO-1, Occludin, and Claudin 3. Control group, L-GLY: low-concentration GLY group (50 mg/kg), H-GLY: high-concentration GLY group (250 mg/kg), H-GLY + Cur: high-concentration GLY (250 mg/kg) + Cur (150 mg/kg). The data are expressed as the mean ± SEM (*n* = 3), and one-way ANOVA was performed, followed by Tukey’s HSD test. Statistically significant compared with controls (* *p* < 0.05; ** *p* < 0.01).

H&E staining results revealed mild structural disorganization in the L-GLY group compared to the control group, and there is also a small amount of inflammatory cell infiltration. In contrast, the H-GLY group exhibited destruction of the alveolar structure in the mammary gland tissue, accompanied by extensive inflammatory cell infiltration within the alveolar lumens. In the H-GLY + Cur group, partial alveolar damage and mild inflammatory infiltration were observed in the mammary tissue (Figure 1A,B).

Furthermore, GLY exposure significantly altered the expression levels of IL-6, IL-1β and TNF-α in mammary relative to the control group (Figure 1C). Similarly, the expressions of tight junction proteins ZO-1, Claudin-3 and Occludin were significantly reduced, suggesting that GLY exposure may compromise the blood–milk barrier and contribute to the development of mastitis. Notably, Cur treatment significantly attenuates GLY-induced tissue injury, suppresses the inflammatory response, and protects the expression of cell-junction proteins, thereby exerting a protective effect on the tissue (Figure 1D).

### 3.2. Transcriptome Sequencing of Damaged Mammary Gland from GLY Exposure

**Figure 2:**
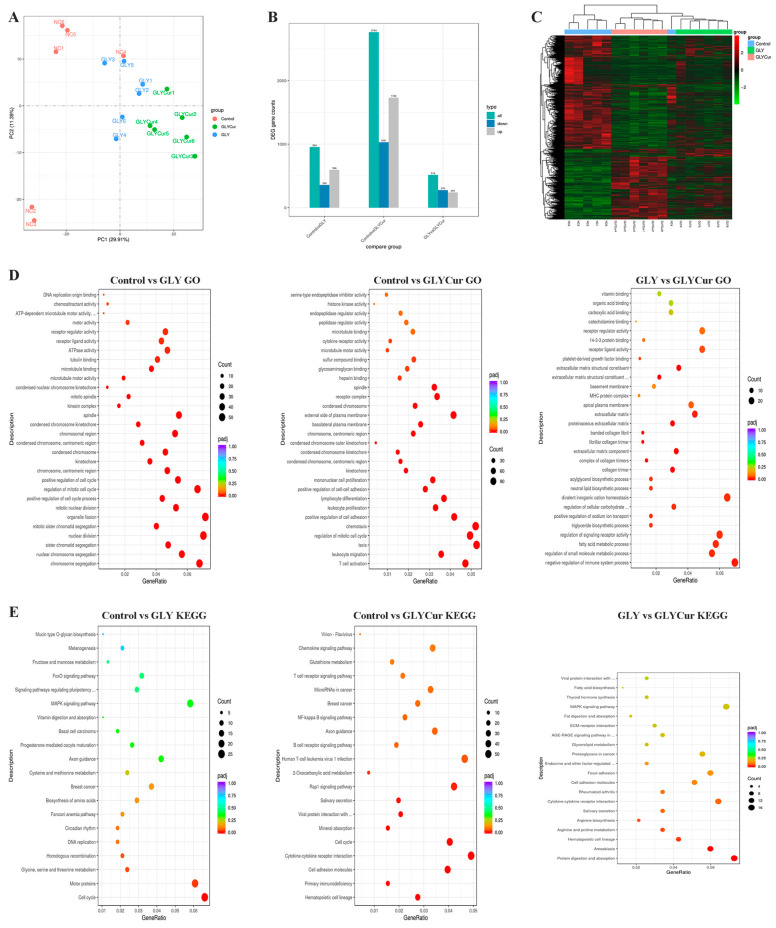
Transcriptome sequencing of mammary tissues from different treatment groups (*n* = 6). (**A**) Principal component analysis. (**B**) Statistical analysis of differentially expressed genes (DEGs). (**C**) Examples of significantly differentially expressed gene heatmap. (**D**) GO enrichment analysis of DEGs in different treatment groups. (**E**) KEGG enrichment analysis of DEGs in different treatment groups.

In order to investigate the mechanism of the response of breast glands to GLY, we conducted RNA sequencing on control, H-GLY and H-GLY + Cur. Principal component analysis (PCA) revealed that the transcriptional patterns of the GLY and GLYCur groups were clearly different from the controls, as shown in Figure 2A. In contrast to the control group, the GLY group showed an increased level of 596 genes and a significantly decreased level of 358 genes. Similarly, the GLYCur group displayed an upregulation of 1733 genes and a downregulation of 1031 genes (Figure 2B). Of these genes, 398 differentially expressed genes were common among the two treated groups. Similarly, the cluster heatmap verified the findings (Figure 2C).

GO enrichment analysis suggested that the differentially expressed genes (DEGs) were predominantly associated with mitotic cell cycle processes in the GLY group. In the GLYCur group, the most prominent biological processes included T cell activation, leukocyte migration and regulation of mitotic cell cycle (Figure 2D). KEGG pathway analysis revealed enrichment of genes associated with the cell cycle, metabolism, DNA replication, and the MAPK signaling pathway in the GLY group (Figure 2E). However, most of the activation pathways in the GLY group were not found in the GLYCur group. The differentially expressed genes were significantly enriched in pathways related to immune response, viral infection, and cellular proliferation. The comprehensive list of specifically regulated genes is provided in Supplementary Table S1. Collectively, these findings indicate that GLY exposure induces extensive transcriptional alterations, whereas curcumin (Cur) may alleviate GLY-induced damage by modulating inflammatory pathways and gene expression patterns.

### 3.3. Effects of GLY and Cur on GMECs Viability

**Figure 3:**
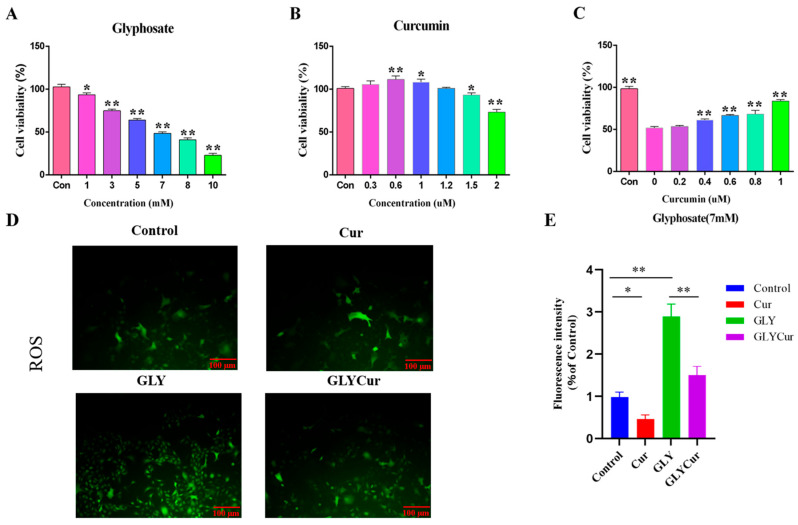
Cur inhibition alleviated GLY-induced cytotoxicity and reactive oxygen species (ROS) levels. (**A**–**C**) Viability of GMECs treated with different concentrations of GLY and Cur for 24 h. Data were normalized by % of the control. (**D**,**E**) ROS levels of different treatment groups. Scale bar is 100 μm. The data are expressed as the mean ± SEM (*n* = 3), and one-way ANOVA was performed, followed by Tukey’s HSD test. Statistically significant compared with controls (* *p* < 0.05; ** *p* < 0.01).

To further explore the toxic mechanism of GLY exposure, a CCK 8 assay was used to assess the potential cytotoxic effect of GLY and Cur on GMECs. We treated GMECs with GLY at multiple concentrations (1, 3, 5, 7, 8 and 10 mM), or Cur at multiple concentrations (0.3, 0.6, 1.0, 1.2, 1.5 and 2.0 uM) after exposure for 24 h. Cell viability decreased significantly when the treatment concentration was within the range of 1 mM to 10 mM GLY (*p* < 0.05). Furthermore, compared to controls, cell viability was increased when the Cur treatment concentration was within the range of 0.6 uM to 1.0 uM (*p* < 0.05). However, cell viability decreased significantly shown at higher concentrations (over 1.5 uM) (Figure 3A,B). These results demonstrate that Cur effectively counteracts GLY-induced cytotoxicity. Additionally, we set 7 mM GLY for the GLY groups and 7 mM GLY and 1 uM Cur for the GLYCur groups for further study (Figure 3C).

Oxidative stress is the most crucial aspect of the toxic effects of pesticides. Reactive oxygen species (ROS) production was measured to explore the molecular mechanism underlying Cur’s protective effect. We assessed ROS generation in the control, Cur, GLY and GLYCur groups. Just as predicted, the GLY group showed a significantly enhanced green fluorescence signal compared to the control group, while the GLYCur group displayed a significantly weaker green fluorescence signal than the GLY groups (Figure 3D,E). These data suggest that Cur mitigates GLY-induced cytotoxicity by suppressing oxidative stress.

### 3.4. Transcriptional Profile of GMECs After Exposure to GLY

**Figure 4:**
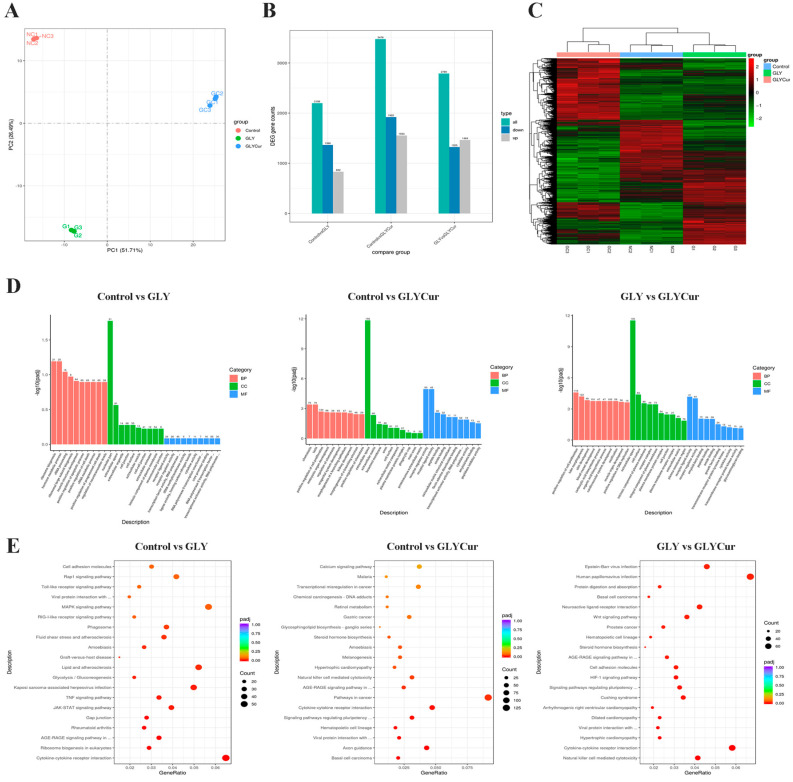
Transcriptome sequencing of GMECs from different treatment groups (*n* = 3). (**A**) Principal component analysis. (**B**) Statistical analysis of differentially expressed genes (DEGs). (**C**) Examples of significantly differentially expressed gene heatmap. (**D**) GO enrichment analysis of DEGs in different treatment groups. (**E**) KEGG enrichment analysis of DEGs in different treatment groups.

To elucidate the molecular mechanisms underlying GLY toxicity and the protective role of Cur, transcriptomic analysis was performed on control, GLY, and GLYCur-treated cells. Principal component analysis (PCA) revealed that the transcriptional patterns of the GLY and GLYCur groups were clearly different from the controls, as shown in Figure 4A. In contrast to the control group, the GLY group showed an increased level of 832 genes and a significantly decreased level of 1366 genes. Similarly, the GLYCur group displayed an upregulation of 1554 genes and a downregulation of 1922 genes (Figure 4B). Hierarchical clustering analysis (Figure 4C) visually confirmed the significant differences in gene expression patterns among these three groups. The GLY group exhibited a distinct transcriptional signature, characterized by widespread gene downregulation. Conversely, the GLYCur group displayed an opposing pattern, where many genes suppressed by GLY recovered to normal or even higher expression levels. Comparative analysis of DEGs between the two models revealed significant alterations in genes associated with ER stress, including GADD45B, DDIT4, TXNIP, DERL3, SOX4, and TRIB2, as well as apoptosis-related genes, such as BNIP3, RNF183, and RNF157 (Supplementary Table S1, Figure S1). Together, these findings directly demonstrate that curcumin can partially counteract the transcriptional dysregulation induced by glyphosate.

GO enrichment analysis revealed that the GLY group significantly enriched biological processes including hormone metabolic process, positive regulation of apoptotic process, positive regulation of cell death, ribosome biogenesis and rRNA processing (Figure 4D). In the GLYCur group, regulation of chemotaxis, positive regulation of cell proliferation, urogenital system development, morphogenesis of a branching epithelium and osteoblast differentiation were significantly altered (Figure 4D). KEGG pathway analysis showed that cytokine–cytokine receptor interaction, ribosome biogenesis in eukaryotes, rheumatoid arthritis, glycolysis/gluconeogenesis, the MAPK signaling pathway and the TNF signaling pathway were activated in the GLY group (Figure 4E). In the GLYCur group, steroid hormone biosynthesis, signaling pathways regulating pluripotency of stem cells, cytokine–cytokine receptor interaction, pathways in cancer, AGE-RAGE signaling pathway in diabetic complications and natural killer cell-mediated cytotoxicity were enriched (Figure 4E). Overall, these results indicate that Cur improves the cytotoxicity caused by GLY by regulating gene expression, thereby restoring the cell’s homeostasis at the transcriptional level.

### 3.5. Cur Alleviated GLY-Induced ER Stress and Ca^2+^ Overload

**Figure 5:**
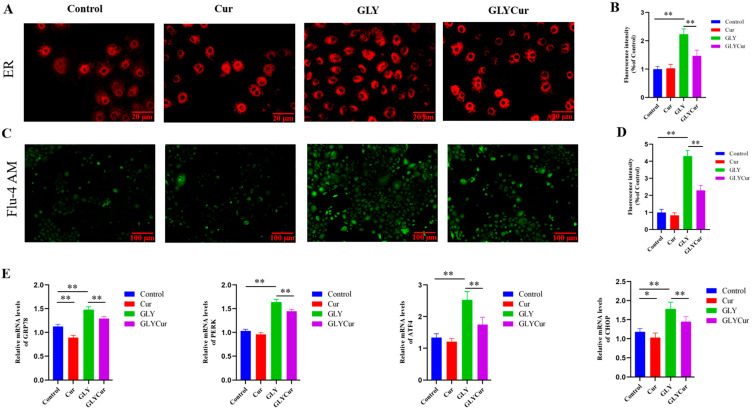
Cur inhibited GLY-induced ER stress and Ca^2+^ overload. (**A**) ER staining after GLY and Cur treatment in GMECs. Scale bar is 20 μm. (**B**) The fluorescence intensity of ER staining. (**C**) Fluorescent images of Ca^2+^ levels in the GLY and Cur treatment groups. Scale is 100 μm. (**D**) Fluorescence intensity of intracellular Ca^2+^ levels. (**E**) Relative mRNA expression of GRP78, PERK, ATF4, and CHOP measured by RT-qPCR (*n* = 3). The data are expressed as the mean ± SEM (*n* = 3), and one-way ANOVA was performed, followed by Tukey’s HSD test. Statistically significant compared with controls (* *p* < 0.05; ** *p* < 0.01).

Reactive oxygen accumulation can disrupt ER homeostasis and lead to ER stress. To investigate the role of endoplasmic reticulum (ER) stress and calcium homeostasis disruption in GLY toxicity, as well as the protective effect of Cur, ER morphology and intracellular Ca^2+^ levels were assessed using fluorescence microscopy and quantitative analysis. In the control and Cur groups, cells exhibited well-organized ER structures with uniform red fluorescence distribution. In contrast, GLY treatment induced marked ER stress, characterized by significantly enhanced and diffuse red fluorescence, indicative of ER swelling or structural disruption. Notably, co-treatment with Cur (GLYCur group) significantly reversed this increase, as evidenced by reduced fluorescence intensity and partial restoration of ER architecture. No significant difference was observed between the Cur alone and control groups, indicating that Cur does not affect ER homeostasis under basal conditions (Figure 5A,B).

ER stress is strongly correlated with Ca^2+^ levels. The control and Cur groups showed weak green fluorescence, representing basal Ca^2+^ levels. GLY treatment triggered severe Ca^2+^ overload, as evidenced by a dramatic increase in green fluorescence intensity. In the GLYCur group, fluorescence intensity was markedly reduced compared with the GLY group, suggesting that Cur mitigates GLY-induced Ca^2+^ accumulation. Consistently with the ER stress results, Cur alone had no significant effect on intracellular Ca^2+^ levels (Figure 5C,D). As shown in Figure 5E, GLY treatment significantly upregulated all four endoplasmic reticulum stress (ERS) markers compared to the control group (*p* < 0.01). In contrast, Cur administration markedly reduced their mRNA levels below control values (*p* < 0.01). Notably, co-treatment with GLYCur partially reversed the GLY-induced upregulation, with mRNA levels of GRP78, IRE1, ATF4, and CHOP being significantly lower than those in the GLY group (*p* < 0.01), although most targets remained elevated relative to the control. Collectively, these results demonstrate that GLY induces ER stress and intracellular Ca^2+^ overload, both of which are effectively alleviated by curcumin, highlighting its protective role against GLY-induced cellular dysfunction.

### 3.6. Cur Alleviated GLY-Induced Apoptosis in GMECs

**Figure 6:**
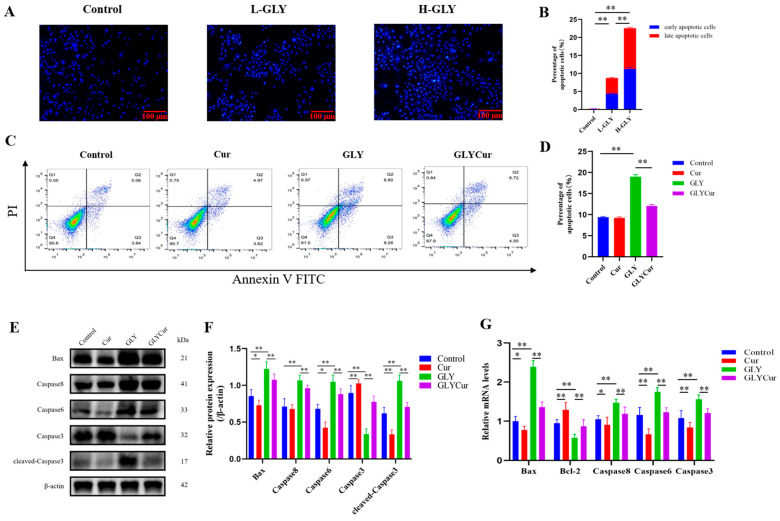
Cur alleviated GLY-induced apoptosis in GMECs. (**A**) Hoechst 33258 staining was used to detect apoptosis cells. Scale bar is 100 μm. (**B**) Graph representing the percentage of early and late apoptosis cells. (**C**) Annexin V-FITC/PI staining after different treatment. (**D**) Graph representing the percentage of apoptotic cells. (**E**,**F**) Protein expression levels of apoptosis-related gene. (**G**) mRNA expression levels of apoptosis-related gene. β-actin was used as a control. The data are expressed as the mean ± SEM (*n* = 3), and one-way ANOVA was performed, followed by Tukey’s HSD test. Statistically significant compared with controls (* *p* < 0.05; ** *p* < 0.01).

To investigate the pro-apoptotic effect of GLY and the anti-apoptotic role of Cur, we conducted a comprehensive analysis of apoptosis. The Hoechst 33258 staining results indicated that in the control group, cell nuclei exhibited regular shapes with uniform chromatin distribution. In contrast, both L-GLY (1 mM) and H-GLY (7 mM) treatments induced typical apoptotic morphological changes, such as chromatin condensation and nuclear fragmentation, which were more pronounced in the H-GLY group. The proportions of early and late apoptotic cells increased significantly in a GLY concentration-dependent manner (Figure 6A,B). Annexin V-FITC/PI double staining followed by flow cytometry was performed to quantify apoptosis (Figure 6C). In the control and Cur groups, the majority of cells were viable, with a very low proportion of apoptotic cells. GLY treatment led to a marked increase in the apoptotic cells, co-treatment with Cur (GLYCur group) significantly reduced the GLY-induced apoptosis rate, indicating that Cur effectively protects cells from GLY-induced apoptosis. Cur treatment alone had no significant effect on cell apoptosis compared with the control group (Figure 6D).

To investigate the effects of Cur, GLY, and their combination (GLYCur) on apoptosis-related signaling, we examined the expression of key apoptotic regulators at both the protein and mRNA levels. Western blot analysis revealed that, relative to the control group, GLY treatment markedly increased the protein levels of the pro-apoptotic protein Bax, caspase-8, caspase-6, caspase-3, and cleaved caspase-3 (Figure 6E). In contrast, Cur treatment significantly suppressed the expression of these proteins. Densitometric quantification confirmed these changes (Figure 6F), showing that the relative protein levels of all examined pro-apoptotic factors were significantly higher in the GLY group than in the control group and significantly lower in the Cur group. At the mRNA level, RT-qPCR analysis (Figure 6G) demonstrated that GLY significantly upregulated Bax, caspase-8, caspase-6, and caspase-3 expression, while downregulating the anti-apoptotic gene Bcl-2. Cur treatment reversed these transcriptional changes, reducing the mRNA levels of Bax and caspase family members and increasing Bcl-2 expression. Notably, co-treatment with GLYCur yielded intermediate phenotypes across all measured parameters, partially attenuating the pro-apoptotic effects of GLY and also blunting the anti-apoptotic actions of Cur. This pattern suggests a potential antagonistic interaction between GLY and Cur in the regulation of apoptosis. Collectively, these results indicate that GLY activates the apoptotic program by upregulating pro-apoptotic factors and downregulating Bcl-2, whereas Cur exerts a protective anti-apoptotic effect.

### 3.7. Curcumin Counteracts GLY-Induced Inflammatory Response via the TNFα/MAPK/STAT3 Axis

**Figure 7:**
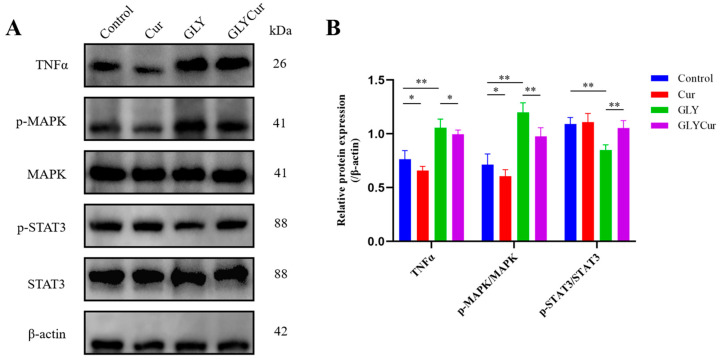
Curcumin attenuates GLY-induced inflammatory response via the TNFα/MAPK/STAT3 axis. (**A**) Representative Western blot images showing the protein levels of TNFα, p-MAPK, MAPK, p-STAT3, STAT3, and β-actin in cells across different treatment groups. (**B**) Quantitative analysis of the relative protein expression levels normalized to β-actin. Data are expressed as mean ± SEM (*n* = 3). Statistical significance was determined by one-way ANOVA followed by Tukey’s HSD test (* *p* < 0.05; ** *p* < 0.01).

Given that the MAPK signaling pathway was enriched in both mouse mammary tissue and GMECs based on transcriptomic sequencing, we next examined the protein expression levels of key molecules in this pathway following GLY exposure using Western blotting (Figure 7A). GLY treatment significantly increased TNFα protein levels and the phosphorylation ratios of both MAPK (p-MAPK/MAPK) and STAT3 (p-STAT3/STAT3) compared with the control group. Conversely, Cur treatment markedly suppressed TNFα expression and p-MAPK activation relative to controls, whereas STAT3 phosphorylation remained comparable to or slightly above baseline. In the combined GLYCur group, TNFα and p-MAPK levels were significantly lower than those in the GLY group, indicating an attenuation of the GLY-induced inflammatory signaling. Total MAPK and STAT3 protein levels were relatively stable across groups, suggesting that the observed effects were driven primarily by changes in phosphorylation states. Collectively, these data indicate that Cur counteracts GLY-triggered pro-inflammatory signaling by modulating the TNFα/MAPK/STAT3 axis.

## 4. Discussion

The introduction of genetically engineered glyphosate-resistant crops in the mid-1990s facilitated the global dissemination of glyphosate applications [40]. The ability of glyphosate to obstruct the shikimic acid pathway is linked to its mode of action [45]. Based on the lack of a shikimate pathway in animals, it has been determined that glyphosate-based herbicides do not present a significant health hazard to both humans and animals. However, recent studies have validated that the presence of glyphosate in the environment may have a greater impact through chronic exposure to lower concentrations of the compound, rather than acute toxicity. There is increased concern regarding the possible negative impacts on animal and human health due to prolonged exposure to glyphosate. As glyphosate’s toxicity has been extensively investigated in various animal models and cell cultures [30], the mammary gland’s susceptibility to this herbicide has received less attention. Mammary epithelial cells (MECs) are of vital significance in the modulation of both milk secretion and the blood–milk barrier (BMB). In the present study, we systematically investigated the toxicological effects of GLY on mammary gland tissue and mammary epithelial cells, as well as the protective potential of Cur. H&E staining results revealed that GLY exposure, particularly at higher doses, caused a pronounced destruction of alveolar structure and extensive inflammatory cell infiltration, a phenotype strikingly similar to that observed in clinical and experimental mastitis [46]. The significant reduction in tight junction proteins (ZO-1, Claudin-3, Occludin) is a critical finding, as it suggests that GLY compromises the blood–milk barrier. The BMB is a selective barrier formed by mammary epithelial cells, and its disruption is a well-established hallmark of mastitis, leading to increased paracellular permeability and allowing pathogens or toxins to enter the milk [47]. The concomitant elevation of pro-inflammatory cytokines IL-6, IL-1β, and TNF-α indicates that GLY not only disrupts physical barriers but also actively instigates an inflammatory cascade. These results confirm that high-dose GLY causes toxic effects in the mammary gland and can trigger mastitis-like conditions. Meanwhile, curcumin can alleviate the breast damage caused by GLY exposure. A limitation of this study is the absence of a curcumin-only group in the in vivo design, which precludes assessment of whether curcumin alone affects mammary morphology, tight-junction proteins, or basal inflammatory tone. Although an in vitro Cur-only group offered partial support, such data cannot substitute for an in vivo control due to systemic differences in metabolism and tissue architecture. Consequently, the protective effects observed in the GLY + Cur group should be interpreted with caution, as they may depend on GLY-induced stress rather than reflect an independent homeostatic action of curcumin. Future studies incorporating a dedicated Cur-only animal group are necessary to resolve this uncertainty.

It is worth noting that the protective effects of curcumin observed in this study occurred in the context of GLY-induced stress, consistently with previous reports demonstrating that curcumin’s cytoprotective actions are typically unmasked under pathological or stress conditions rather than in healthy, unchallenged tissues [48,49,50]. Additionally, it must be acknowledged that both the in vivo doses (50 and 250 mg/kg/day) and the in vitro concentration (7 mM) used here are considerably higher than typical real-world exposure levels; for comparison, human urinary glyphosate levels are generally in the μg/L range, and dietary intake is estimated to be far below mg/kg/day [28,29]. The in vivo doses were selected to establish a clear toxic phenotype and to probe the protective mechanism of curcumin under conditions of robust mammary gland damage, whereas the in vitro concentration was chosen to induce measurable cellular stress and ER/UPR activation within a short experimental window. While the H&E findings at higher in vivo doses revealed mastitis-like pathology, the actual glyphosate concentrations reached in mammary tissue are likely in the micromolar range, substantially lower than the 7 mM applied to cultured cells. This dose–context gap underscores that the in vitro model primarily informs pathway-level mechanisms rather than directly replicating in vivo exposure conditions. Future studies incorporating dose–response analyses at lower, more physiologically relevant concentrations will be necessary to bridge this gap and to determine whether similar inflammatory and structural alterations occur under chronic low-dose scenarios.

To investigate the potential mechanisms of glyphosate-induced mammary toxicity, we performed RNA sequencing of both mammary tissue and GMECs. In vivo, GLY predominantly enriched pathways related to the cell cycle, DNA replication, and MAPK signaling, indicative of a strong proliferative stress response. In contrast, GMECs exposed to GLY exhibited a markedly different transcriptional landscape dominated by widespread gene suppression and enrichment of apoptotic processes, ribosome biogenesis, and cytokine–cytokine receptor interactions. The comprehensive list of specifically regulated genes is provided in Supplementary Table S1. This discrepancy between in vivo and in vitro responses likely reflects not only the complex multicellular environment of the mammary gland, where stromal–immune–epithelial crosstalk modulates GLY toxicity, but also the fundamental difference in exposure conditions: the tissue concentrations achieved in vivo via oral gavage are subject to absorption, distribution, and metabolism, and are not directly comparable to the sustained millimolar GLY exposure used in cell culture. Consequently, the protective effects of curcumin observed in vivo may involve systemic or metabolic factors that are not recapitulated in the in vitro model, and the relative contributions of different stress pathways—oxidative stress, ER stress, and apoptosis—may differ between the two systems. Given that the MAPK signaling pathway is highly conserved across mammalian species [51], we employed mouse mammary tissue from pregnant mice to evaluate developmental roles in vivo and goat lactating GMECs to assess secretory functions under a shared regulatory framework. We recognize that both the cross-species design and the concentration gap represent limitations of the current study, and future investigations employing a single species with matched exposure paradigms are warranted to validate these findings.

Previous reports have shown that exposure to glyphosate at any life stage reduces cell viability [52,53]. In the results of the cell viability assay, GLY exposure markedly decreased cell viability while the addition of curcumin significantly increased cell viability. The treatment concentration of GLY ranging from 1 to 10 mM substantially decreased cell viability. Moreover, cell viability was found to be substantial and increased at 0.6 to 1.0 uM concentrations of Cur relative to the control group. In contrast, a substantial reduction in cell viability was noted at exceeding 1.5 uM concentrations. The findings indicated that GLY dose-dependently caused cytotoxicity and decreased cell viability in GMECs. Furthermore, GLY treatment of GMECs was shown to induce apoptosis of the cells.

Mitochondria are one of the primary contributors to ROS production within cells. These organelles experience significant changes in their function when exposed to OS conditions. The production of ROS will result in the breakdown of the mitochondrial membrane potential, leading to prolonged mitochondrial dysfunction and eventual damage to the cell [54]. According to our transcriptome data, GLY significantly suppressed the expression of key antioxidant genes, including SOD1, CAT, and GPX4. The ER is exquisitely sensitive to redox imbalance. Our data establish that GLY induces acute ER stress and intracellular Ca^2+^ overload in mammary epithelial cells, characterized by disrupted ER morphology, elevated cytosolic Ca^2+^, and transcriptional upregulation of the UPR markers GRP78, IRE1, ATF4, and CHOP [55]. This ER stress response is functionally linked to apoptosis, as GLY concurrently activated the mitochondrial apoptotic pathway, evidenced by Bax and caspase upregulation, Bcl-2 suppression, and increased Annexin V-positive cells. The observation that both ER stress and apoptosis were partially but significantly reversed by Cur points to a mechanistic model in which GLY provokes Ca^2+^-dependent ER stress that, in turn, triggers the intrinsic apoptotic cascade, and Cur protects cells by restoring ER and Ca^2+^ homeostasis. Notably, the GLYCur combination consistently yielded intermediate phenotypes rather than complete rescue across ER stress markers and apoptotic indicators. This pattern suggests a functional antagonism whereby Cur counteracts a subset of GLY-triggered upstream signals—likely those mediated by oxidative stress and Ca^2+^ dysregulation—but cannot fully intercept other GLY-induced perturbations, such as direct effects on ER membrane integrity or additional stress-sensing pathways. The reciprocal dampening of both agents’ individual effects further indicates that GLY and Cur do not simply neutralize each other chemically but engage overlapping stress–apoptosis signaling nodes in a competing manner. In conclusion, our findings demonstrate that ER stress is a central mediator of GLY cytotoxicity and that Cur exerts its protective effect, at least partly, by preserving ER and Ca^2+^ homeostasis, thereby attenuating the downstream apoptotic program. The limited reversibility observed underscores the complexity of GLY toxicity and the need for caution when extrapolating from acute high-dose in vitro models. Future studies employing UPR-specific inhibitors, caspase blockers, or genetic modulation of Bcl-2 family members will be necessary to establish causal relationships and evaluate whether this antagonistic interplay translates to chronic, low-concentration exposure scenarios in vivo.

The integration of our in vivo and in vitro transcriptomic data consistently pointed to the activation of the MAPK and TNF signaling pathways. Our Western blot validation provides a mechanistic synthesis: GLY activates the TNFα/MAPK/STAT3 inflammatory signaling axis in mammary epithelial cells, evidenced by increased TNFα expression and elevated phosphorylation of MAPK and STAT3. This is consistent with the ability of GLY to trigger oxidative stress and downstream pro-inflammatory cascades. MAPK signaling is a classical driver of inflammation, promoting the transcription of pro-inflammatory cytokines (IL-6, IL-1β). STAT3 can have both pro- and anti-inflammatory roles; however, reduced p-STAT3 is often associated with impaired tissue repair and persistent inflammation. The simultaneous activation of pro-inflammatory MAPK and suppression of potentially protective STAT3 signaling creates a powerful inflammatory environment [56]. Curcumin markedly suppressed TNFα levels and MAPK phosphorylation, aligning with its known anti-inflammatory action, yet it did not reduce STAT3 phosphorylation; instead, p-STAT3 remained at or slightly above control levels. This differential regulation suggests that curcumin may uncouple MAPK and STAT3 signaling, possibly preserving a STAT3-mediated survival program while dampening MAPK-driven inflammation. In the combined treatment, TNFα and p-MAPK were significantly lower than in the GLY group, demonstrating that curcumin attenuates GLY-induced inflammatory signaling. The stable total protein levels further highlight that altered phosphorylation, rather than protein abundance, underlies these effects. Together, these data place the TNFα/MAPK/STAT3 axis as a key effector of GLY-induced stress and suggest that curcumin counteracts this pathway primarily by targeting TNFα and MAPK activation. However, these observations remain correlative; functional studies using MAPK or STAT3 inhibitors are necessary to confirm causality and dissect the precise role of STAT3 in this antagonistic interaction.

## 5. Conclusions

In conclusion, our findings demonstrate that exposure to glyphosate (GLY), a widely used herbicide, induces dose-dependent mammary gland injury characterized by acinar destruction, inflammatory infiltration, and disruption of the blood–milk barrier. In vitro experiments further confirm that GLY triggers oxidative stress, endoplasmic reticulum (ER) stress, and intracellular Ca^2+^ overload, which collectively activate the mitochondrial apoptotic pathway and the TNFα/MAPK/STAT3 inflammatory axis. Notably, co-treatment with curcumin (Cur) effectively reverses these pathological and molecular alterations by partially attenuated GLY-induced transcriptomic alterations, inhibiting reactive oxygen species (ROS) production, alleviating ER stress, preventing apoptosis, and suppressing the inflammatory signaling cascade. Collectively, these results establish glyphosate as a direct mammary toxicant capable of eliciting mastitis-like pathological changes, and highlight curcumin—a naturally derived antioxidant—as an effective dietary intervention for mitigating glyphosate-associated mammary toxicity.

## Figures and Tables

**Table 1:** Primer sequences.

Gene	Accession Numbers	Target Species	Primer Sequences (5′ → 3′)
β-actin	NM_007393.5	Mouse	F: GATCTGGCACCACACCTTCT R: CAGAGGCATACAGGGACAGC
IL-6	NM_031168.2	Mouse	F: AACGATGATGCACTTGCAGA R: TCTCTCTGAAGGACTCTGGCT
IL-1β	NM_008361.4	Mouse	F: AATCTCGCAGCAGCACATCAAC R: TCCACGGGAAAGACACAGGTAG
TNFα	NM_013693.3	Mouse	F: ACGTGGAACTGGCAGAAGAGG R: TGAGAAGAGGCTGAGACATAGGC
ZO-1	NM_011442.5	Mouse	F: TGTAGACGATCATCCACCCAAAGC R: ATTAGGCAGAGCACCATCAGAAGG
Occludin	NM_016674.4	Mouse	F: CCTCTGACCTTGAGTGTGGATGAC R: TCCTCTTGCCCTTTCCTGCTTTC
Claudin3	NM_009902.4	Mouse	F: GGCGGCTCTGCTCACCTTAG R: CGTACAACCCAGCTCCCATCTC
β-actin	NM_001009884.1	Goat	F: TCCTCCCTGGAGAAGAGCTAC R: GGTAGTTTCGTGAATGCCGC
GRP78	XM_005687138.3	Goat	F: GTTGGGGTGTTCAAGAACGG R: GTCGAAGACTGTGTTCTCAGGA
PERK	XM_018058668.1	Goat	F: CAGCAAAGAGGAGCCCAGAATG R: CAGACACAGTAGGTTCGGAAGAAG
ATF4	XM_018046529.1	Goat	F: GCAGTGAAGTGGTTATCTCTGAAGG R: TGTCGTTATCTGAGTGGGCATCC
CHOP	XM_005680285.3	Goat	F: TCTGGCTTGGCTTACTGAGGAG R: TTCTGGGTCTTCCTTGGTCTTCC
Bax	NM_001287077.1	Goat	F: CCAAGAAGCTGAGCGAGTGTCTG R: GTGTCCACGGCTGCGATCATC
Bcl2	XM_018039337.1	Goat	F: TGGATGACCGAGTACCTGAACCG R: TGCCTTCAGAGACAGCCAGGAG
Caspase 3	XM_018058668.1	Goat	F: GTGGTATTGAGACAGACAGTGGTTC R: CCAGGTGCTGTAGAATATGCGTAC
Caspase 6	XM_018058668.1	Goat	F: GGACGCAGCCTCGGTTTATACAC R: TCCCCAGCATCTCACACAAATCTTG
Caspase 8	XM_018058668.1	Goat	F: GACCGACTCAGAACAGATGGAAGC R: CCCAGCAGAAAGTCAGCCTCATC

**Table 2:** Antibody information for Western blots.

Gene	Host Species	Manufacturer	Product Number	Dilution Ratio
β-actin	Mouse	Beyotime, Shanghai, China	AA128	1:1000
BAX	Rabbit	Beyotime, Shanghai, China	AB026	1:500
Caspase 3	Rabbit	Affinity, Changzhou, China	AF6311	1:1000
Cleaved Caspase 3	Rabbit	Affinity, Changzhou, China	AF7022	1:1000
Caspase 6	Rabbit	Beyotime, Shanghai, China	AF1927	1:500
Caspase 8	Rabbit	Beyotime, Shanghai, China	AF1243	1:500
TNFα	Rabbit	Abways, Shanghai, Chin	CY8873	1:1000
P-MAPK	Rabbit	Abways, Shanghai, China	BY0109	1:500
MAPK	Rabbit	Abways, Shanghai, China	CY5262	1:1000
P-STAT3	Rabbit	Abways, Shanghai, Chin	CY5291	1:500
STAT3	Rabbit	Abways, Shanghai, Chin	CY5292	1:1000
HRP-labeled Goat Anti-Rabbit IgG (H + L)	Rabbit	Beyotime, Shanghai, China	A0208	1:1000
HRP-labeled Goat Anti-Mouse IgG (H + L)	Mouse	Beyotime, Shanghai, China	A0216	1:1000

## Data Availability

Data are available in a publicly accessible repository. The original data presented in this study are openly available in the Sequence Read Archive (SRA) under accession number PRJNA1472956.

## Supplementary Materials

The following supporting information can be downloaded at https://www.mdpi.com/article/10.3390/antiox15070893/s1. Figure S1: Venn diagram showing the overlap of differentially expressed genes between in vivo and in vitro RNA‑seq datasets. Table S1: the differentially expressed genes (DEGs) in each dataset (Goat vs. Mouse).

## Abbreviations

The following abbreviations are used in this manuscript:

AMPA	Aminomethylphosphonic acid
BMB	Blood–milk barrier
Cur	Curcumin
DEGs	Differentially expressed genes
ER	Endoplasmic reticulum
ERS	Endoplasmic reticulum stress
FAO	Food and Agriculture Organization of the United Nations
GLY	Glyphosate
GMECs	Goat mammary epithelial cells
IARC	International Agency for Research on Cancer
IBD	Inflammatory bowel disease
PCA	Principal component analysis
ROS	Reactive oxygen species
WHO	World Health Organization

## References

[1] Peillex C., Pelletier M. (2020). The impact and toxicity of glyphosate and glyphosate-based herbicides on health and immunity. J. Immunotoxicol..

[2] Ganesan S., Keating A.F. (2023). Maternal impacts of pre-conceptional glyphosate exposure. Toxicol. Appl. Pharmacol..

[3] Ren Y.L., Chen K., Li Y., Jia Y.Z., Wang H., Wang L. (2026). Environmental glyphosate exposure compromises sperm quality in mice by impairing acrosome biogenesis via GOLPH3-mediated golgiphagy. J. Hazard. Mater..

[4] Newman M.M., Hoilett N., Lorenz N., Dick R.P., Liles M.R., Ramsier C., Kloepper J.W. (2016). Glyphosate effects on soil rhizosphere associated bacterial communities. Sci. Total Environ..

[5] Barnett J., Josephson J.K., Yuzbashian E., Verdugo A., McComb C.J., Bandy M.L., Ghosh S., Letef C., Murch S.J., Jung M.M. (2025). Prenatal exposure to dietary levels of glyphosate disrupts metabolic, immune, and behavioral markers across generations in mice. Sci. Total Environ..

[6] Romualdo G.R., Valente L.C., de Souza J.L., Rodrigues J., Barbisan L.F. (2023). Modifying effects of 2,4-D and Glyphosate exposures on gut-liver-adipose tissue axis of diet-induced non-alcoholic fatty liver disease in mice. Ecotoxicol. Environ. Saf..

[7] Masood M.I., Naseem M., Warda S.A., Tapia-Laliena M., Rehman H.U., Nasim M.J., Schäfer K.H. (2021). Environment permissible concentrations of glyphosate in drinking water can influence the fate of neural stem cells from the subventricular zone of the postnatal mouse. Environ. Pollut..

[8] Szepanowski F., Kleinschnitz C., Stettner M. (2019). Glyphosate-based herbicide: A risk factor for demyelinating conditions of the peripheral nervous system. Neural Regen. Res..

[9] European Food Safety Authority (2015). Conclusion on the peer review of the pesticide risk assessment of the active substance glyphosate. EFSA J..

[10] Myers J.P., Antoniou M.N., Blumberg B., Carroll L., Colborn T., Everett L.G., Hansen M., Landrigan P.J., Lanphear B.P., Mesnage R. (2016). Concerns over use of glyphosate-based herbicides and risks associated with exposures: A consensus statement. Environ. Health.

[11] Mills P.J., Caussy C., Loomba R. (2020). Glyphosate excretion is associated with steatohepatitis and advanced liver fibrosis in patients with fatty liver disease. Clin. Gastroenterol. Hepatol..

[12] Shu S.G., Chen X., Ren J.W., Yu X.Y., Hao Z., Yu Y.Q. (2025). Glyphosate Induces Anxiety-Like Behaviors in Mice via Activating NLRP3-Mediated Hippocampal Microglia Pyroptosis. J. Appl. Toxicol..

[13] Ford B., Bateman L.A., Gutierrez-Palominos L., Park R., Nomura D.K. (2017). Mapping proteome-wide targets of glyphosate in mice. Cell Chem. Biol..

[14] Gasnier C., Dumont C., Benachour N., Clair E., Chagnon M.C., Séralini G.E. (2009). Glyphosate-based herbicides are toxic and endocrine disruptors in human cell lines. Toxicology.

[15] Thongprakaisang S., Thiantanawat A., Rangkadilok N., Suriyo T., Satayavivad J. (2013). Glyphosate induces human breast cancer cells growth via estrogen receptors. Food Chem. Toxicol..

[16] Kwiatkowska M., Huras B., Bukowska B. (2014). The effect of metabolites and impurities of glyphosate on human erythrocytes (in vitro). Pestic. Biochem. Physiol..

[17] Mesnage R., Ibragim M., Mandrioli D., Falcioni L., Tibaldi E., Belpoggi F., Brandsma I., Bourne E., Savage E., Mein C.A. (2022). Comparative toxicogenomics of glyphosate and roundup herbicides by mammalian stem cell-based genotoxicity assays and molecular profiling in Sprague-Dawley rats. Toxicol. Sci..

[18] Larsen K., Lifschitz A., Fernandez San Juan R., Virkel G. (2022). Metabolic stability of glyphosate and its environmental metabo lite (aminomethylphosphonic acid) in the ruminal content of cattle. Food Addit. Contam. Part A.

[19] Nova P., Calheiros C.S.C., Silva M. (2020). Glyphosate in Portuguese adults—A pilot study. Environ. Toxicol. Pharmacol..

[20] Dominguez A., Brown G.G., Sautter K.D., de Oliveira C.M., de Vasconcelos E.C., Niva C.C., Bartz M.L., Bedano J.C. (2016). Toxicity of AMPA to the earthworm Eisenia andrei Bouche, 1972 in tropical artificial soil. Sci. Rep..

[21] Gomez A.L., Altamirano G.A., Alcaraz M.R., Montemurro M., Schierano-Marotti G., Oddi S.L., Culzoni M.J., Muñoz-de-Toro M., Bosquiazzo V.L., Kass L. (2023). Mammary gland development in male rats perinatally exposed to propiconazole, glyphosate, or their mixture. Environ. Toxicol. Pharmacol..

[22] Meftaul I.M., Venkateswarlu K., Dharmarajan R., Annamalai P., Asaduzzaman M., Parven A., Megharaj M. (2020). Controversies over human health and ecological impacts of glyphosate: Is it to be banned in modern agriculture. Environ. Pollut..

[23] Mansour S.A., Mossa T.H. (2010). Oxidative damage, biochemical and histopathological alterations in rats exposed to chlorpyrifos and the antioxidant role of zinc. Pestic. Biochem. Physiol..

[24] Mattson M.P. (2014). Neuronal life-and-death signaling, apoptosis, and neurodegenerative disorders. Antioxid. Redox Signal..

[25] Edinger A.L., Thompson C.B. (2004). Death by design: Apoptosis, necrosis and autophagy. Curr. Opin. Cell Biol..

[26] Battaglin W.A., Meyer M.T., Kuivila K.M., Dietze J.E. (2014). Glyphosate and Its Degradation Product AMPA Occur Frequently and Widely in U.S. Soils, Surface Water, Groundwater, and Precipitation. J. Am. Water Resour. Assoc..

[27] Niemann L., Sieke C., Pfeil R., Solecki R. (2015). A critical review of glyphosate findings in human urine samples and comparison with the exposure of operators and consumers. J. Verbraucherschutz Leb..

[28] Conrad A., Schröter K.C., Hoppe H., Rüther M., Pieper S., Kolossa G.M. (2017). Glyphosate in German adults—Time trend (2001 to 2015) of human exposure to a widely used herbicide. Int. J. Hyg. Environ. Health.

[29] Kruger M., Schledorn P., Schrödl W., Hoppe H.W., Lutz W., Shehata A.A. (2014). Detection of Glyphosate Residues in Animals and Humans. J. Environ. Anal. Toxicol..

[30] Van B., He M.M., Shin K., Mai V., Jeong K.C., Finckh M.R., Morris J.G. (2018). Environmental and health effects of the herbicide glyphosate. Sci. Total Environ..

[31] Tarazona J.V., Court M.D., Tiramani M., Reich H., Pfeil R., Istace F., Crivellente F. (2017). Glyphosate toxicity and carcinogenicity: A review of the scientific basis of the European Union assessment and its differences with IARC. Arch. Toxicol..

[32] Wafi A.M., Hong J., Rudebush T.L., Yu L., Hackfort B., Wang H., Schultz H.D., Zucker I.H., Gao L. (2019). Curcumin improves exercise performance of mice with coronary artery ligation-induced HFrEF: Nrf2 and antioxidant mechanisms in skeletal muscle. J. Appl. Physiol..

[33] Kotha R., Luthria D. (2019). Curcumin: Biological, Pharmaceutical, Nutraceutical, and Analytical Aspects. Molecules.

[34] Mirzaei H., Masoudifar A., Sahebkar A., Zare N., Sadri N., Rashid B., Mehrabian E., Mohammadi M., Mirzaei H.R., Jaafari M.R. (2018). MicroRNA: A novel target of curcumin in cancer therapy. J. Cell. Physiol..

[35] Yang D., Wang L., Zhang L., Wang M., Li D., Liu N., Liu D., Zhao M., Yao X. (2024). Construction, characterization and bioactivity evaluation of curcumin nanocrystals with extremely high solubility and dispersion prepared by ultrasound-assisted method. Ultrason. Sonochem..

[36] Carro-Rodríguez J., Ibáñez-Cervantes G., Cárdenas-Rodríguez N., Ignacio-Mejía I., Albores-Méndez E.M., Pardo-Pacheco B.R., Fernández-Sánchez V., Balboa-Verduzco A.M., Adame C., Lara-Padilla E. (2026). Microbiota-Gut-Brain Axis Disruption, Neuroinflammation, and Potential Antioxidant-Based Treatments in Metabolic Diseases. Antioxidants.

[37] Lee W.H., Loo C.Y., Khor P.Y., Gnanaraj C., Koh C.P., Leong C.R., Dua K., Yeung S., Cheong K.L. (2026). Curcumin Synergistically Sensitizes Multidrug-Resistant Lung Cancer to Doxorubicin Through Ferroptosis-Associated Oxidative Stress. Antioxidants.

[38] Zhu H.T., Bian C., Yuan J.C., Chu W.H. (2014). Curcumin attenuates acute inflammatory injury by inhibiting the TLR4/MyD88/NF-kappaB signaling pathway in experimental traumatic brain injury. J. Neuroinflamm..

[39] Pandur E., Gulyás-Fekete G., Kulcsár G., Huber I. (2025). Synthetic Cyclic C5-Curcuminoids Increase Antioxidant Defense and Reduce Inflammation in 6-OHDA-Induced Retinoic Acid-Differentiated SH-SY5Y Cells. Antioxidants.

[40] Gianessi L.P. (2005). Economic and herbicide use impacts of glyphosate-resistant crops. Pest Manag. Sci..

[41] Bartholomew S.K., Winslow W., Sharma R., Pathak K.V., Tallino S., Judd J.M., Leon H., Turk J., Pirrotte P., Velazquez R. (2024). Glyphosate exposure exacerbates neuroinflammation and Alzheimer’s disease-like pathology despite a 6-month recovery period in mice. J. Neuroinflamm..

[42] Guo X.X., Xu Y., Geng R.X., Qiu J., He X.Y. (2022). Curcumin Alleviates Dextran Sulfate Sodium-Induced Colitis in Mice Through Regulating Gut Microbiota. Mol. Nutr. Food Res..

[43] Wang Z.H., Liu S.J., Cheng Z.F., Li F., Bu Q.Q., Zhang L., Song Y.X., An X.P. (2024). Endoplasmic reticulum stress exacerbates microplastics-induced toxicity in animal cells. Food Res. Int..

[44] Li F., Wang Z.H., Luo M.H., Hu J.X., Wang H.F., He Y.L., Zhang L., Cao B.Y., An X.P., Song Y. (2025). Tea polyphenols attenuate glufosinate-induced breast injury by reducing endoplasmic reticulum stress and autophagy. J. Hazard. Mater..

[45] Panzacchi S., Tibaldi E., De Angelis L., Falcioni L., Giovannini R., Gnudi F., Iuliani M., Manservigi M., Manservisi F., Manzoli I. (2025). Carcinogenic effects of long-term exposure from prenatal life to glyphosate and glyphosate-based herbicides in Sprague-Dawley rats. Environ. Health.

[46] Zanardi M.V., Schimpf M.G., Gastiazoro M.P., Milesi M.M., Muñoz-de-Toro M., Varayoud J., Durando M. (2020). Glyphosate-based herbicide induces hyperplastic ducts in the mammary gland of aging Wistar rats. Mol. Cell. Endocrinol..

[47] Altamirano G.A., Masat E., Rivera O., Alarcón R., Dioguardi G., Muñoz-de-Toro M., Luque E.H., Kass L. (2023). Postnatal exposure to a glyphosate-based herbicide interferes with the development and growth of the mammary gland of pre-pubertal Ewe lambs. Chemosphere.

[48] Khuwaja G., Moni S.S., Alam M.F., Makeen H.A., Zafar S., Ashafaq M., Alhazmi H., Najmi A., Sayed S.F., Iqubal S.M.S. (2024). Curcumin nanogel and its efficacy against oxidative stress and inflammation in rat models of ischemic stroke. Nanomedicine.

[49] Dai P., Xu L.Y., Zhang P., Liang Z., Chu Y.H., Yu Z.Q., Cao L., Sun P., Li X. (2025). Protective effects of curcumin on epileptic rodent models by alleviating oxidative stress and inflammation: A meta-analysis and mechanism exploration. Front. Pharmacol..

[50] Yang C.K., Zhu Q.W., Chen Y.B., Ji K., Li S.H., Wu Q., Pan Q.Q., Li J. (2024). Review of the Protective Mechanism of Curcumin on Cardiovascular Disease. Drug Des. Dev. Ther..

[51] Shi T., Niepel M., McDermott J.E., Gao Y., Nicora C.D., Chrisler W.B., Markillie L.M., Petyuk V.A., Smith R.D., Rodland K.D. (2016). Conservation of protein abundance patterns reveals the regulatory architecture of the EGFR-MAPK pathway. Sci. Signal..

[52] Mehtiyev T., Karaman E.F., Ozden S. (2023). Alterations in cell viability, reactive oxygen species production, and modulation of gene expression involved in mitogen-activated protein kinase/extracellular regulating kinase signaling pathway by glyphosate and its commercial formulation in hepatocellular carcinoma cells. Toxicol. Ind. Health.

[53] Da K.N., Cappellaro L.G., Ueda C.N., Rodrigues L., Remor A.P., Martins R.D., Latini A., Glaser V. (2020). Glyphosate-based herbicide impairs energy metabolism and increases autophagy in C6 astroglioma cell line. J. Toxicol. Environ. Health A.

[54] Mesnage R., Ferguson S., Brandsma I., Moelijker N., Zhang G., Mazzacuva F., Caldwell A., Halket J., Antoniou M.N. (2022). The surfactant co-formulant POEA in the glyphosate-based herbicide RangerPro but not glyphosate alone causes necrosis in Caco-2 and HepG2 human cell lines and ER stress in the ToxTracker assay. Food Chem. Toxicol..

[55] Chukwubueze F., Reyes C.D.G., Chávez-Reyes J., Solomon J., Sandilya V., Sahioun S., Marichal-Cancino B.A., Mechref Y. (2025). Multi-Omics Alterations in Rat Kidneys upon Chronic Glyphosate Exposure. Biomolecules.

[56] Zhang M.Y., Sun X.W., Yu X., Xu L., Zhang X.R., Zhang R.N., Lu H., Tang C.L., Wu Z.H., Zhu J. (2025). Limosilactobacillus reuteri regulates gut microbiota and increases the effective metabolite luteolin to inhibit MAPK/STAT3 signaling pathway to alleviate allergic rhinitis. Front. Microbiol..

