# Supplementary material for: Curcumin Attenuates Glyphosate-Induced Mammary Toxicity via Suppression of ER Stress and the TNFα/MAPK/STAT3 Axis
Source: Antioxidants (Basel). 2026 Jul 19;15(7):893. doi: 10.3390/antiox15070893 (PMC13406013; doi:10.3390/antiox15070893)
Supplement: Supplementary file 1 [file antioxidants-15-00893-s001.zip › Supplementary Material.pdf]

Supplementary Material

Curcumin Attenuates Glyphosate-Induced Mammary Toxicity via Suppression of ER Stress and the TNFα/MAPK/STAT3TNFα-MAPK-STAT3 Axis

Yonglong He, Hanbing Yan, Zesheng Gan, Ziwei Cheng, Binyun Cao, Jiangang Wang * and Xiaopeng An *

College of Animal Science and Technology, Northwest A&F University, Yangling, Shaanxi 712100, P.R. China.

Yonglong He: heyonglong96@nwafu.edu.cn

Hanbing Yan: yanhanbing1128@163.com

Zesheng Gan: 11569352928@qq.com

Ziwei Cheng: 11251724927@qq.com

Binyun Cao: caobinyun@126.com

Jiangang Wang: wangjiangang236@126.com

Xiaopeng An: anxiaopengdky@163.com

Corresponding Author: Jiangang Wang, Xiaopeng An

Phone number: 13772162131, 029-87092102

E-mail: wangjiangang236@126.com, anxiaopengdky@163.com

Address: College of Animal Science and Technology, Northwest A&F University, Yangling, Shaanxi 712100, P.R China

To gain mechanistic insights, we examined the transcriptional profiles of genes associated with endoplasmic reticulum (ER) stress and apoptosis across the experimental groups. In the Control vs. GLY comparison, stress-responsive genes such as GADD45B, CRYAB, SOX4, and TRIB2 were significantly upregulated, suggesting the initiation of cellular stress following glyphosate exposure. Notably, a distinct set of ER stress-related modulators, including DDIT4, TXNIP, DERL3, and NUPR1, were identified as differentially expressed in the GLY vs. GLYCur group, indicating that curcumin co-treatment effectively mitigated the glyphosate-induced unfolded protein response (UPR). Regarding apoptosis, the GLY vs. GLYCur comparison revealed significant changes in pro-apoptotic effectors such as BNIP3, RNF183, and RNF157, while the anti-apoptotic marker BCL2 was concomitantly altered. Furthermore, the differential regulation of MAPK signaling components, including MAP2K6, MAP3K6, and multiple dual-specificity phosphatases (DUSP4, DUSP5, DUSP6, DUSP10), points to the complex involvement of the p38/JNK cascade in mediating stress-induced cell death. The comprehensive list of specifically regulated genes is provided in Supplementary Table S1. Collectively, these findings indicate that high-dose glyphosate exposure triggers ER stress-mediated apoptotic pathways in mammary tissue, which can be effectively suppressed by curcumin intervention.

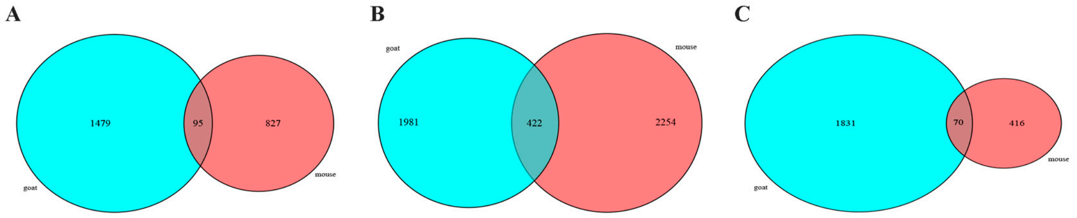

Figure S1 The differentially expressed genes (DEGs) in each dataset (Goat vs. Mouse). (A) Control vs GLY. (B) Control vs GLYCur. (C) GLY vs GLYCur.
